# Long-lived weight-reduced αMUPA mice show higher and longer maternal-dependent postnatal leptin surge

**DOI:** 10.1371/journal.pone.0188658

**Published:** 2017-11-30

**Authors:** Mariel Pinsky, Maayan Rauch, Atallah Abbas, Adi Sharabi-Nov, Snait Tamir, Roee Gutman

**Affiliations:** 1 Unit of Integrative Physiology (LIP), Laboratory of Human Health and Nutrition Sciences, MIGAL—Galilee Research Institute, Kiryat Shmona, Israel; 2 Laboratory of Human Health and Nutrition Sciences, MIGAL—Galilee Research Institute, Kiryat Shmona, Israel; 3 Department of Nutritional Sciences, Faculty of Sciences and Technology, Tel-Hai College, Upper Galilee, Israel; 4 Research Wing, Ziv Medical Center, Zefat, Israel; 5 Department of Animal Sciences, Faculty of Sciences and Technology, Tel-Hai College, Upper Galilee, Israel; Hospital Infantil Universitario Nino Jesus, SPAIN

## Abstract

We investigated whether long-lived weight-reduced αMUPA mice differ from their wild types in postnatal body composition and leptin level, and whether these differences are affected by maternal-borne factors. Newborn αMUPA and wild type mice had similar body weight and composition up to the third postnatal week, after which αMUPA mice maintained lower body weight due to lower fat-free mass. Both strains showed a surge in leptin levels at the second postnatal week, initiating earlier in αMUPA mice, rising higher and lasting longer than in the wild types, mainly in females. Leptin level in dams’ serum and breast milk, and in their pup’s stomach content were also higher in αMUPA than in the WT during the surge peak. Leptin surge preceded the strain divergence in body weight, and was associated with an age-dependent decrease in the leptin:fat mass ratio—suggesting that postnatal sex and strain differences in leptin ontogeny are strongly influenced by processes independent of fat mass, such as production and secretion, and possibly outside fat tissues. Dam removal elevated corticosterone level in female pups from both strains similarly, yet mitigated the leptin surge only in αMUPA–eliminating the strain differences in leptin levels. Overall, our results indicate that αMUPA’s postnatal leptin surge is more pronounced than in the wild type, more sensitive to maternal deprivation, less related to pup’s total adiposity, and is associated with a lower post-weaning fat-free mass. These strain-related postnatal differences may be related to αMUPA’s higher milk-borne leptin levels. Thus, our results support the use of αMUPA mice in future studies aimed to explore the relationship between maternal (*i*.*e*. milk-borne) factors, postnatal leptin levels, and post-weaning body composition and energy homeostasis.

## Introduction

Postnatal perturbations in pups physiology often lead to long-term adverse effects–a phenomenon known as “the developmental origins of health and disease” [1]. Specifically, postnatal perturbations in circulating levels of the hormone leptin were found to be associated with altered energy homeostasis [2,3]. Leptin is secreted mainly by white adipose [4], as well as by mammary glands [5], placenta [6], and gastric epithelial cells [7]. In adults, leptin’s serum level is correlated with body fat and its main role is in signaling a negative energy balance [8]. However, this role of leptin is absent in pre-weaning rodents [2,3,9,10]. Postnatal leptin level in rodents shows a transient increase peaking at the 2^nd^ postnatal week, termed leptin surge [11–13]. This surge is related, to a varying degree, to leptin’s mRNA expression levels in white and brown fat and in gastric epithelial cells, as well as to total fat mass [7,13–17]. Leptin surge was also suggested to be related to dam’s diet and milk-borne leptin levels [5,17–20], while others suggested that pups leptin level is rather related to the pup’s nutritional status [14,21–24]. Irrespective of leptin’s origin, previous studies have established that neonatal leptin acts as a developmental factor involved in rewiring hypothalamic circuits and peripheral organs involved in energy homeostasis [25–29]. Indeed, perturbation of postnatal leptin level or its signaling in *ad lib* fed mice alter post-weaning energy homeostasis, as reviewed by [30]. However, the causality between leptin surge level and post-weaning body weight is hard to prove [30,31], as these phenotypes seem to be associated with mode of leptin delivery, pups’ sex and diet, and most importantly, with maternal diet during gestation and lactation (cf. [14,22,32–38] and [39–43].

In this study we further investigate these issues using αMUPA (alpha murine urokinase-type plasminogen activator, uPA) mice and their WTs [44,45]. αMUPA mice carry as a transgene the cDNA-encoding for the murine uPA linked to the enhancer–promoter region of the α-crystalline gene [46]. uPA is an extracellular serine protease implicated in fibrinolysis, tissue remodeling [47], brain plasticity [48,49], and neuroprotection [50]. As expected from the transgenic promoter, expression of the transgenic uPA was detected in the ocular lens, as well as ectopically in the brain [51]. As we and others have shown, αMUPA mice show longer lifespan and altered energy homeostasis compared with their FVBN/N wild type (WT) mice, including lower body weight and food intake along with higher leptin levels [44,45,52]. The reduced food intake phenotype in αMUPA has been described also in two transgenic lines [51,53], thus pointing to uPA, the product of the transgenic expression, as the primary causative factor. Yet, the direct link between the transgenic expression, leptin levels, and metabolic changes is not clear. Nevertheless, and as suggested previously [44], the transgenic effect is likely to be developmental, similarly to the impressive remodeling effect recently described for aMUPA’s developing incisor teeth [54].

Notably, the divergence in body-weight growth curves between aMUPA and its WT occurs at the third postnatal week [45,52], the age at which leptin surge ends in other strains [11–13]. This result prompted our working hypothesis that αMUPA mice have an altered postnatal leptin ontogeny compared with their WT’s. Thus, the main goal of this study was to test this hypothesis by looking into both strain (αMUPA vs. WT) and sex (male vs. female) differences in postnatal ontogeny of leptin level (explored in the first experiment). At the same time, we explored the postnatal ontogeny of body composition (fat mass, FM; fat free mass, FFM; and fat %), and its association with leptin ontogeny. Based on these results, we investigated in subsequent experiments the contribution of maternal (i.e., milk-borne) factors to the observed strain differences in leptin surge. This last goal was fulfilled in the second and third experiments by: a, depriving pups of their dams for four hours (second experiment) and comparing their leptin levels with those of non-deprived pups (from the first experiment); and b, measuring maternal serum and milk-borne leptin levels, as well as their pup’s stomach content and serum leptin levels, during the peak of its surge (third experiment). For the second and third experiments we used only female pups, who showed greater strain differences in the first experiment. The results of these three experiments enabled further speculation about the role of leptin in determining the αMUPA phenotype.

## Results

### *αMUPA*’s preweaning lower body weight is due to lower fat free mass

**Fig 1:**
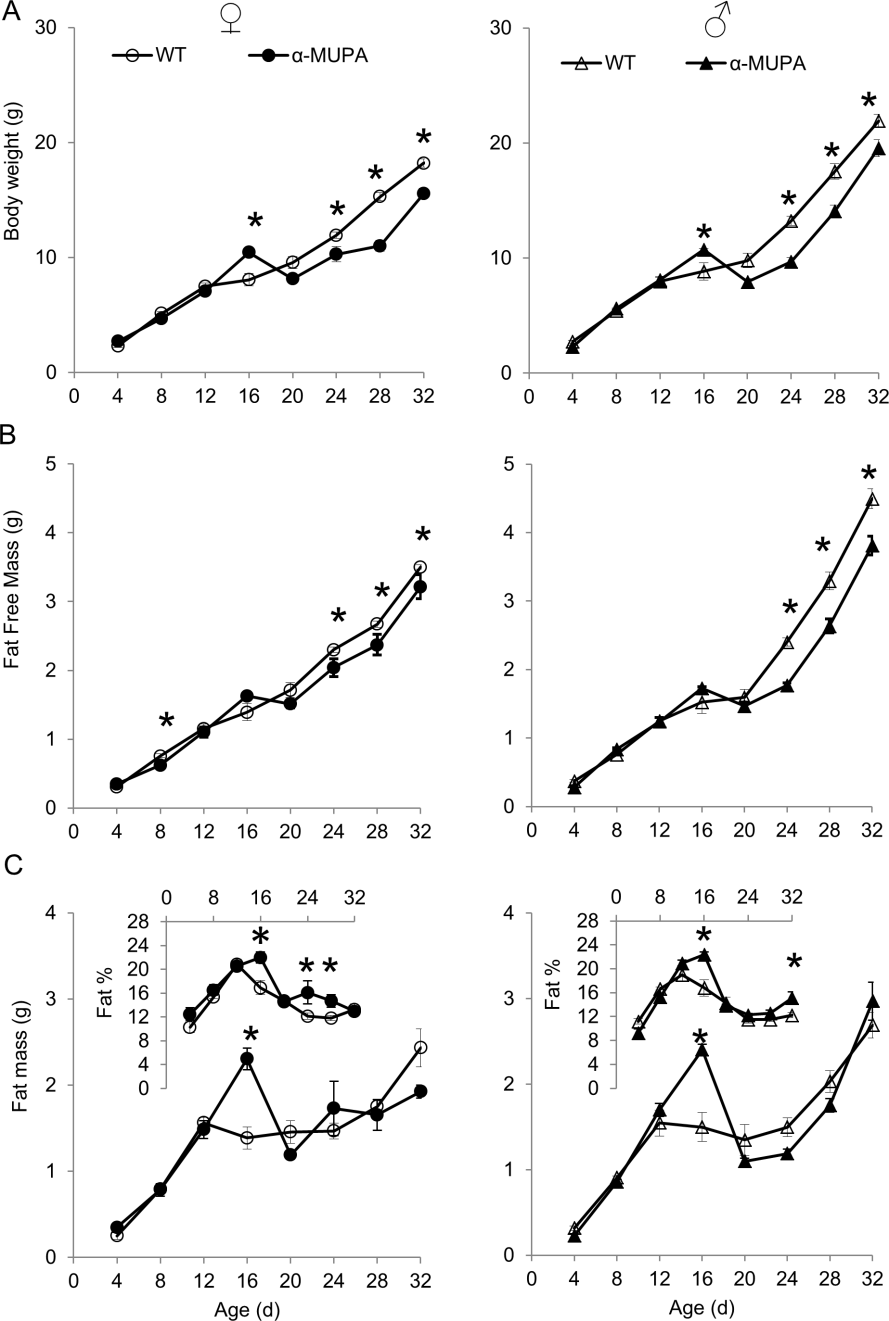
αMUPA’s body weight is lower than that of their WT as from the 3^rd^ postnatal week due to lower fat free mass. Each time point represents the mean ± s.e.m. of 6 to 22 mice (see S2 Table for details). Pups were sampled every four days, following four hours of chow deprivation (first experiment), and were weaned at day 24 (P24). (A) Body weight growth curves of males (right) and females (left). (B) Postnatal ontogeny of fat-free mass measured by full body chemical extraction. (C) Postnatal ontogeny of fat mass measured by full body chemical extraction. Insert of C. Postnatal ontogeny of fat percentage, calculated from body composition data. Bars with different letters are significantly different (*P*<0.05, based on two-ways ANOVA followed by a post-hoc test). *, *P*<0.05 by post hoc analysis following one-way ANOVA of all four mice groups.

Body weight and composition, measured from the fourth day after birth (P4) to P32, were affected by strain, sex, age, and their interactions (S1 Table). Newborn mice (P4 to P12) had similar body weight and composition regardless of sex or strain (Fig 1). During this 8-day period, the increase in body weight resulted mainly from an increase in fat mass (FM, Δ of ca. 550% vs. fat free mass Δ of ca. 350%; *P*<0.001, Fig 1B and 1C). Thereafter, the increase in WT’s FM was halted until weaning (P24) while FFM (fat free mass) increased continuously (Fig 1B and 1C). In contrast, FM and FFM of αMUPA mice increased continuously until P16, followed by a sharp decrease in FM towards P20 (Fig 1B and 1C). As such, body weight of αMUPA mice became noticeably lower than their WT by P20 in both sexes, and significantly so by P24 (Fig 1A). This strain divergence in body-weight growth curves (Fig 1A) was associated with αMUPA’s significantly lower levels of FFM (rather than FM) as from P24 (Fig 1B and 1C). Preceding the onset of body-weight divergence, αMUPA’s body weight at P16 was actually significantly higher than its WT’s (Fig 1A), driven by a higher FM rather than FFM (Fig 1B and 1C), explaining about 80% of the strain difference in dry mass at P16.

Newborn (P4) mice had about 10% fat mass. Fat percentage doubled during the second postnatal week, reaching the highest level documented in this study, and thereafter decreased throughout weaning (Fig 1C, insert). Although αMUPA mice had significantly lower body weight than WT mice as from P24, their fat percentage tended to be similar or higher throughout most of their growth (Fig 1C, insert). Sexual dimorphism in body weight (males being heavier than females) was significant as from P24 for WT and P28 for αMUPA mice. This sexual dimorphism in body weight was mainly due to dimorphism in FFM rather than fat mass.

### *αMUPA* mice have a higher and longer postnatal leptin surge

**Fig 2:**
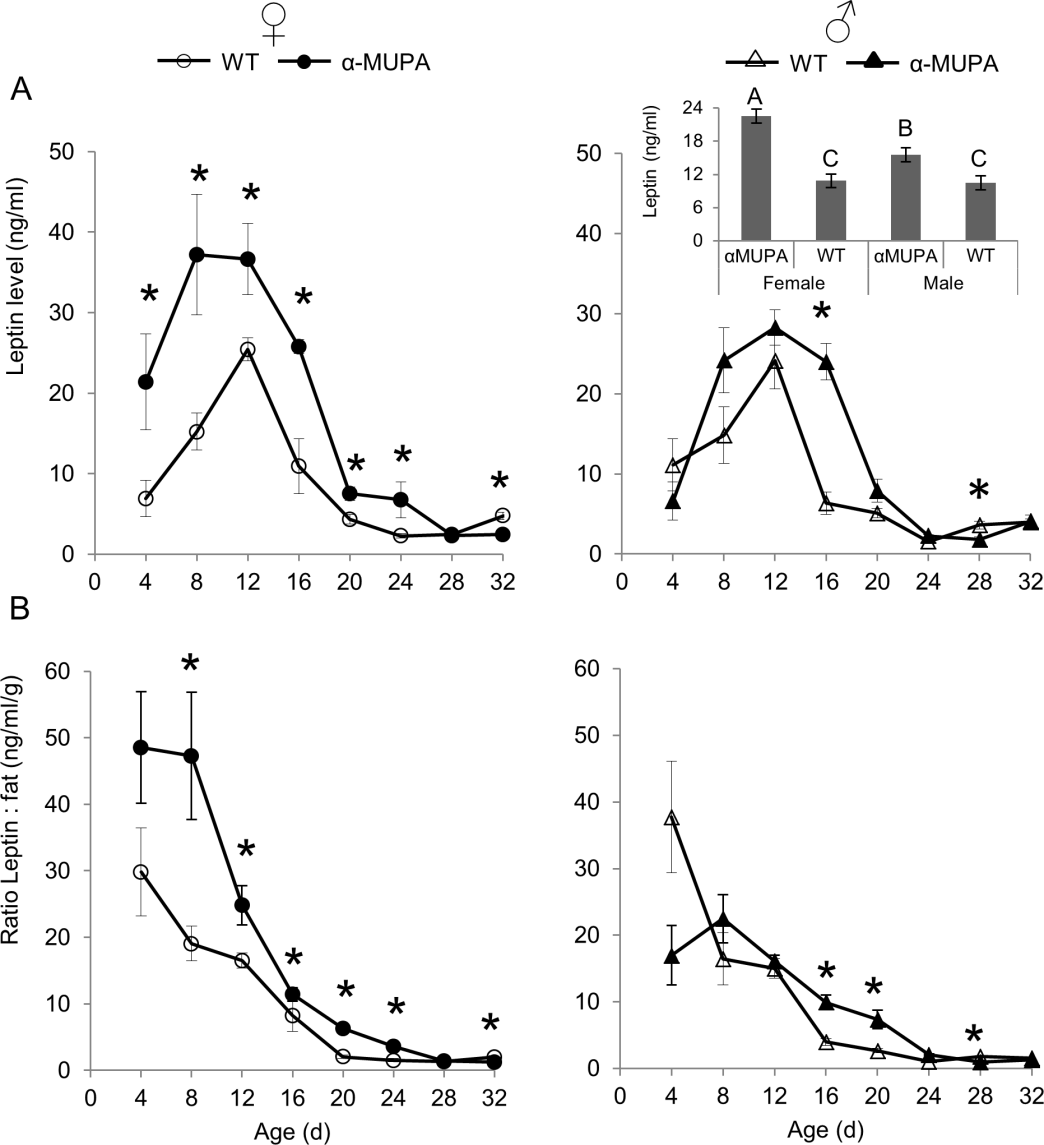
αMUPA mice show a higher and longer postnatal leptin surge due to a higher leptin:Fat mass ratio. Each time point represents the mean ± s.e.m. of 5 to 17 mice (see S2 Table for details). Pups were sampled every four days, following four hours of chow deprivation (first experiment), and were weaned at day 24 (P24). (A) Postnatal circulating leptin level measured using ELISA kit (R&D Systems Inc.). Insert of A. Mean P4-P24 leptin level. Bars with different letters are significantly different (*P*<0.05, based on two-ways ANOVA followed by a post-hoc test). (B) Leptin:FM ratio. *, *P*<0.05 by post hoc comparison following one-way ANOVA of all four mice groups.

Leptin levels of WT and αMUPA mice at P4-P32 were significantly affected by age, strain, and sex, as well as the interactions of strain with sex and strain with age (S1 Table). αMUPA female mice had higher leptin levels than their male littermates as early as P4 (*P* = 0.06, Fig 2A), and much higher than female WT mice (Fig 2A). WT mice, on the other hand, showed no sexual dimorphism in leptin level at P4 (Fig 2A). During the following pre-weaning period (P4-24), leptin levels of all four mice groups increased by 1.7- to 4.3-fold by P12, and decreased to below P4 levels by weaning (P24, Fig 2A). However, the leptin surge of female αMUPA mice rose to higher levels than that of female WT mice, yet declined to similar or lower levels at older age (Fig 2A). On average, during the pre-weaning period, αMUPA mice had significantly higher leptin levels than their WT (indicating a greater surge), with αMUPA females showing greater surge than their male littermates, while WTs lacked sexual dimorphism (Fig 2A, insert).

Notably, following the peak in leptin levels, or alternatively the reduction in its level below a certain threshold (approx. 24 mg per ml), there was a temporary attenuation in body growth (Fig 1A and Fig 2A). For example, in WT mice, leptin peaked at P12, followed by a steep decrease in leptin level and an attenuation of body-weight growth between P12 and P20, especially in males (Fig 1A and Fig 2A). As for αMUPA mice, both sexes showed a longer leptin surge, and accordingly the attenuation of their body-weight growth occurred 4 days later (between P16 and P20), preceding the strain divergence in body weight (Fig 1A and Fig 2A). In fact, αMUPA mice of both sexes showed a temporal reduction in their body weight between P16 to P20, initiating the divergence that became significant at P24 (Fig 1A).

### Age-dependent leptin:Fat mass ratio better explains leptin’s postnatal surge than total fat mass

Researchers have debated about the extent to which postnatal leptin level is related to fat mass (FM) [2,15]. Our data show that the relationship between FM and leptin (Fig 1C and Fig 2A) could be divided into three phases: The first phase (up to P12), in which both leptin level and fat mass increased; the second phase (P12 to P24), in which leptin decreased sharply in both WT and αMUPA, while FM leveled in WT but peaked sharply around P16 in αMUPA; and the third phase (from P24 onwards), in which FM increased sharply while leptin increased moderately in both strains. In order to test the relative contribution of fat mass to leptin level, we applied a multiple regression analysis on the P4-P24 surge data, using leptin as the response variable, and strain, sex, age, and FM as predictors. All variables were found to have a significant effect on leptin (R^2^ = 0.45 in final model, Table 1). Age had the highest degree of importance in the model, as suggested by the *β* coefficients, fat mass was the second highest predictor of leptin levels, whereas strain and sex were the least important, albeit still significant (Table 1). We also calculated the Pearson correlation between FM and leptin (pooled within strain, sex, and age). The correlation was positive, yet with a very small effect size (r = 0.173, p<0.05). Using age as a control variable resulted in a significantly larger effect (Sobel’s test, p<0.001, z = 3.95; r = 0.529, p<0.001, S3 Table), confirming that the association of leptin with fat mass is age-dependent. Measuring the same correlations in each mouse group yielded four positive correlations further confirming the importance of age as a leptin predictor within each group of mice.

**Table 1:** Multiple regression of leptin on strain, gender, age, fat mass, and fat-free mass.

Factor	Β	S.E.	*β*
Strain (α-MUPA)	5.83	1.47	0.22***
Gender (female)	4.49	1.46	0.17***
Fat mass (gr)	12.34	1.37	0.62***
Age (days)	-1.40	0.13	-0.73***
F_(4,186)_		37.49***	
R^2^		0.45	

***p<0.001

As age had the highest degree of importance in the regression model (Table 1) and improved the Pearson correlation of leptin and fat, we also applied the same multiple regression analysis on each age group separately (Table 2). FM was a sole predictor of leptin level only at P4 and post-weaning (>24). During the rest of the surge (P8-24), the contribution of FM was either non-significant (P16-P20) or dependent on strain and sex (P8-12 and P24). This analysis reveals that FM *per se* cannot be considered as the main predictor of postnatal leptin levels during the surge, and does not explain well the differences between αMUPA and WT mice or the sex differences within αMUPA mice.

**Table 2:** Multiple regression of leptin on age, fat mass, strain, and gender, carried out separately for each age group.

Age (days)	*β* coefficients			R^2^	p-value
	Strain	Sex	Fat mass		
4	0.02	0.09	0.85***	0.77	<0.001
8	0.48**	0.40*	0.35*	0.40	0.01
12	0.33*	0.34*	0.50**	0.47	0.001
16	0.54*	0.20	0.30	0.59	<0.001
20	0.74**	-0.10	0.30	0.31	0.01
24	0.40**	0.17	0.63***	0.66	<0.001
28	-0.24	-0.03	0.47**	0.35	0.01
32	-0.30	0.25	0.69***	0.47	0.01

*p<0.05

**P<0.01

***p<0.001

Looking at the ratio of leptin level and FM across age better illustrates the weak relationship between FM and leptin level and the relatively strong influence of age (Fig 2B). The leptin:FM ratio was affected significantly by age, sex, and strain, as well as their interactions (S1 Table). The highest ratio for WT mice was at P4, decreasing sharply thereafter and leveling at the end of the surge (P24) without sexual dimorphism (Fig 2B). αMUPA mice followed that same pattern with two exceptions. First, the reduction in ratio was postponed to P8 and the leveling was postponed to P28. Second, αMUPA females had a higher ratio than their male littermates across several age groups (Fig 2B). Most importantly, the leptin:FM ratio of αMUPA female mice was significantly higher than that of WT females across several age groups (Fig 2B), and αMUPA male mice had a significantly higher leptin:FM ratio than WT males during the third postnatal week (P16 and P20; Fig 2B). Thus, changes in the leptin:FM ratio correspond well with the differences in circulating leptin across the three phases identified above, between the strains as well as between the sexes. This correspondence suggests that the αMUPA’s altered leptin ontogeny is strongly affected by age-dependent processes unrelated to fat mass.

### Maternal-borne factors explain *αMUPA*’s higher and longer postnatal leptin surge

**Fig 3:**
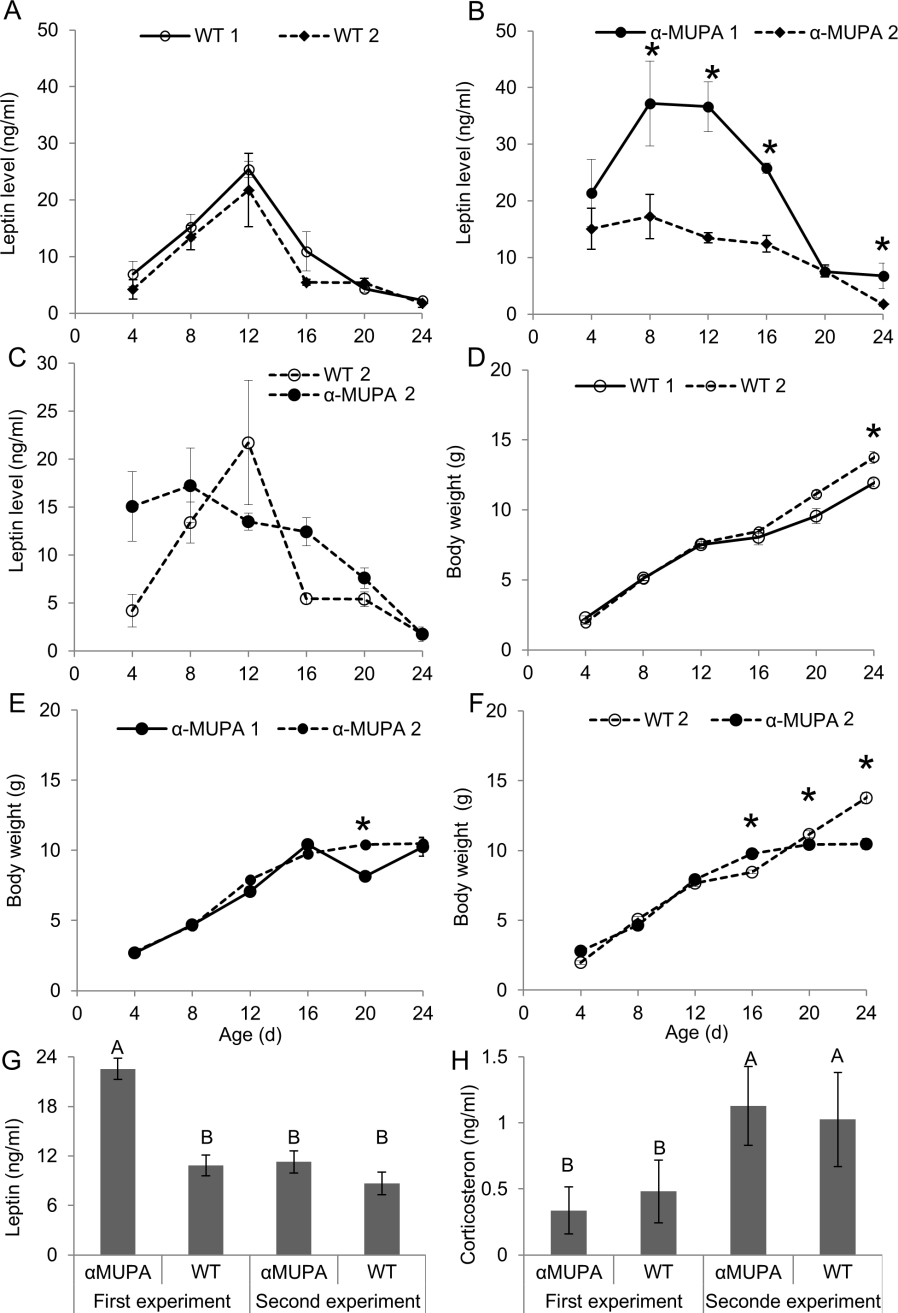
Dams removal eliminates strain differences in postnatal leptin surge by decreasing αMUPA’s leptin levels in a stress- independent manner. Each time point represents the mean ± s.e.m. of 6 to 22 mice, females only. Pups were sampled every four days, following four hours of chow deprivation (first and second experiments) and dam deprivation (second experiment only). Leptin and corticosterone levels were measured using ELISA kits (R&D Systems Inc.). (A and B) Postnatal circulating leptin level of dam-deprived and non-deprived female WT (left) and αMUPA (right) mice. (C) Postnatal circulating leptin levels of dam-deprived female αMUPA and WT mice. (D) Postnatal body weight growth curves of dam-deprived and non-deprived female WT mice. (E) Postnatal body weight growth curves of dam-deprived and non-deprived female αMUPA mice. (F) Postnatal body weight growth curves of dam-deprived female αMUPA and WT mice. (G) Mean P4-P24 leptin level of dam-deprived and non-deprived female αMUPA and WT mice. (H) Corticosterone P12 level of dam-deprived and non-deprived female αMUPA and WT mice. Bars with different letters are significantly different (*P*<0.05, based on two-ways ANOVA followed by a post-hoc test). *, *P*<0.05 by post hoc comparison following one-way ANOVA of all four mice groups.

The goal of the second and third experiments was to test whether strain differences in postnatal leptin levels were affected by maternal-borne factors, an hypothesis that is still highly debatable [2]. Circulating leptin levels of WT female pups were unaffected by maternal deprivation, peaking during the second postnatal week and decreasing to P4 levels around weaning, in accord with leptin surge in the first experiment (Fig 3A). In contrast, maternal deprivation of αMUPA female mice was accompanied by significantly lower leptin levels during the surge (P8, P12, P16, and P24; S4 Table), compared with the non-deprived αMUPA female pups, eliminating the strain differences found in the first experiment (Fig 3B, 3C and 3G). These differences in αMUPA’s leptin levels between deprived and non-deprived pups were not explained by αMUPA’s body weight, as the growth curves of both strains were largely similar in both experiments (Fig 3D and 3E). In accord with the first experiment, αMUPA female pups had lower body weight as from P20 than the WT, while the opposite was true at P16 (Fig 3F).

Finally, and in accord with studies on other rodent strains ([24,55,56] but c.f. [13]), maternally deprived P12 pups from both strains showed significantly higher corticosterone plasma levels compared with non-deprived same-sex pups (Fig 3H). However, under both manipulations (experiments 1 and 2), αMUPA’s corticosterone levels resembled that of their WT’s (Fig 3H). This implies that the sensitivity of αMUPA female pups to stress induced by food restriction, with and without maternal separation, is similar to that of their WT. And more importantly, that αMUPA’s attenuated surge under maternal deprivation is not to likely be related to lower (or higher) stress. To sum, these results indicate that postnatal leptin level in αMUPA mice are likely to be influenced by maternal factors independent of stress.

**Fig 4:**
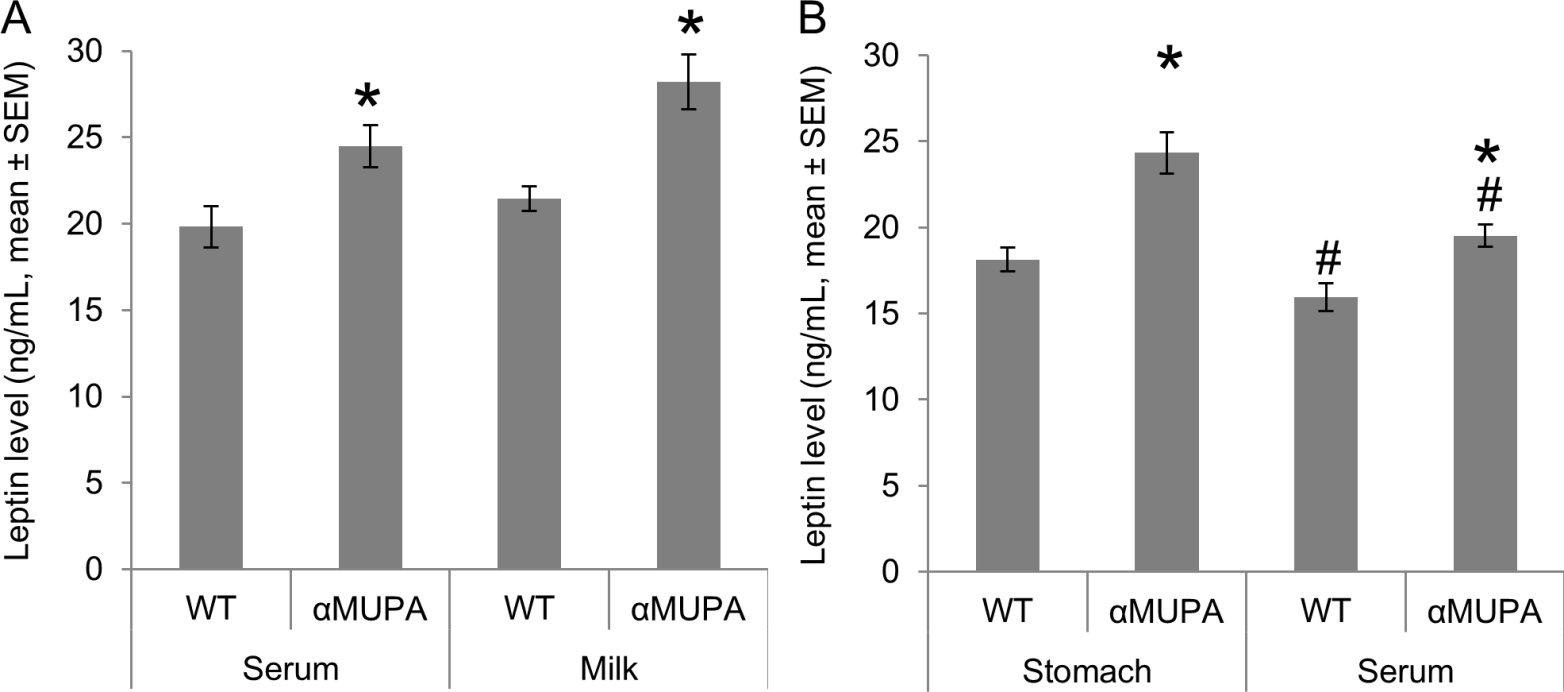
Leptin levels are higher in lactating aMUPA dams’ serum and milk, as well as in their P12 pups’ serum and stomach content. Bars represent the mean ± s.e.m. of 6 dams per strain (A) and their female pups (B), one female pup per dam, all sampled at P12. Dams' serum and milk samples (A) were obtained 4 hours after their separation from their pups. Milk secretion was encouraged by subcutaneous oxytocin injection (2 IU/kg) administered 15 min before sampling. Pups' serum and stomach content (B) were sampled 30 min after they were reunited with their dams. Leptin level was measured using ELISA kit (R&D Systems Inc.). *, P<0.05 between strains, by Mann-Whitney non-parametric test. #, P<0.05 within strains, by Wilcoxon non-parametric Test.

In order to further explore the effect of maternal factors on postnatal leptin levels, we measured leptin levels in dam’s serum and milk in both strains as well as in their female pup’s serum and stomach content at P12, during the peak of the leptin surge (Fig 2A). As expected from our previous studies [44,45], serum leptin level was higher in αMUPA dams than in their WT’s (Fig 4A). In addition, we found here that leptin level was also higher in the breast milk of αMUPA dams than in their WT’s at P12 (Fig 4A). These strain differences in leptin levels were also found in the stomach content and serum level of the female pups (Fig 4B). These results imply, albeit not conclusively, that higher serum leptin levels in aMUPA pups can be attributed (at least to some extent) to gastrointestinal absorption of milk-borne leptin.

## Discussion

Our findings show that the well-known postnatal leptin surge in mice peaks higher and lasts longer in the transgenic αMUPA mouse compared with its wild type strain, and more so in females than males. This surge precedes a divergence in body weight growth curves between the two strains due largely to αMUPA’s lower fat-free mass. In addition, we find that the strongest predictor of postnatal leptin level was age rather than fat mass, through its effect on the leptin:FM ratio, suggesting that postnatal differences within and between strains and sexes in leptin ontogeny are strongly influenced by processes independent of fat mass and possibly outside fat tissues. Moreover, serum and milk leptin levels are higher in αMUPA dams and correspondingly in their pups’ stomach content and serum, compared with their wild types. Maternal deprivation reduces the leptin surge only in αMUPA’s pups, eliminating the strain differences in postnatal leptin levels. Finally, maternal deprivation resulted in a higher corticosterone levels in both strains to the same extent, implying that the aforementioned phenotypes are not likely to be related to differences in stress responsiveness. Thus, these results support the link between overexpression of uPA protein and higher leptin levels, which may be involved in αMUPA’s altered phenotype via maternal-related developmental factors.

### *αMUPA*’s lower body weight is due to pre-weaning lower fat-free mass

The results of this study recapitulate the original αMUPA female study [52], as well as our latest αMUPA male study [45], showing that αMUPA’s lower body weight phenotype emerges before weaning, around the 3^rd^ post-natal week. The novelty in the current study is that newborn αMUPA mice have a similar body composition as their WTs up to their third postnatal week; that αMUPA’s body weight (mainly FFM) is significantly higher at P16; and that the subsequent lower body weight of αMUPA mice is due to their lower FFM.

Based on the literature, the increase in mice body weight up to P10 is driven mainly by an increase in FM (increasing fat percentage), followed by an increase in FFM up to P20-21 (decreasing fat percentage), and a henceforward increase in both FM and FFM [16,57]. We have recently shown that middle-aged αMUPA female mice have lower fat percentage than their WTs [44], but this issue of body composition has not yet been addressed in αMUPA male mice, and has not been explored along the postnatal development trajectory of either strain. Our current results show that αMUPA and WT mice from both sexes follow the aforementioned pattern of postnatal ontogeny of body composition, indicating that overexpression of uPA does not alter this pattern. In addition, both strains follow the same sexual dimorphism in body weight and composition as seen in many other rodent strains, involving higher FM and FFM in males [58,59]. However, in accord with our previous study regarding body weight [45], the onset of sexual dimorphism in both body weight and body composition was delayed in αMUPA mice, emerging at P28 instead of P24. Therefore, it may by concluded that the overexpression of uPA in αMUPA mice delays the ontogeny of sexual dimorphism, but does not alter its general trajectory.

αMUPA mice were originally considered an animal model for longevity induced by caloric restriction [51]. However, Froy and colleagues [44] have shown that mature αMUPA mice have the metabolic profile of satiated animals. In addition, other studies have shown that in under-nourished pups body weight decreases due to lower FM [16,35,60] rather than lower FFM–as is the case in αMUPA mice. Therefore, in accord with Froy et al. [44], our finding that αMUPA differs from the wild type in its FFM rather than FM suggests that their lower body weight is not due to a nutritional shortage.

The biochemical mechanism underlying the unique properties of αMUPA (i.e., the lower FFM) may involve lower serum levels of insulin-like growth factor 1 (IGF-1) [51]. IGF-1 is a circulating growth factor produced mainly by the liver but also by bone marrow and skeletal muscles [61]. IGF-1 knock-out mice show reduced body weight and length, resulting from reduced skeletal and muscle mass [61]. Transgenic mice with reduced IGF-1 serum levels show the combination of lower body weight and longer life span [62]. Accordingly, our long-lived weight-reduced mature αMUPA female mice have lower levels of IGF-1 than their WTs [51]. It would be interesting to see in future studies whether neonatal αMUPA mice also have a lower IGF-1 level compared with their WTs, as it may explain, at least partially, their consequent lower body (fat-free) mass.

### *αMUPA* mice show a higher and longer postnatal leptin surge

Higher leptin level has been suggested as a key regulator in αMUPA’s phenotype [44,45]. Our main goal was to study leptin’s postnatal ontogeny in both sexes of αMUPA and FVB/N (WT) mice, and to test the hypothesis that leptin surge would be different in the weight-reduced long-lived αMUPA strain. Our results show that the patterns of higher leptin levels reported for mature αMUPA hold for neonatal male and female mice as well, and are age- and sex-dependent. Similarly to other rodent models [11–13], postnatal circulating leptin levels of newborn αMUPA and WT mice from both sexes surge around the 2^nd^ postnatal week. However, this leptin surge is significantly greater (both longer and higher) in αMUPA, particularly in females. Previous studies have demonstrated convincingly that neonatal leptin signaling is involved in long-term energy homeostasis [30]. Yet, they also demonstrate the difficulty in establishing causality between neonatal leptin levels and susceptibility to obesity. The effect of increased leptin surge, or decreased leptin signaling, has been inconsistent even when using similar manipulations. For example, inconsistencies were found when neonatal leptin levels were elevated by repeated injections (cf. [32] and [33,34,39], which were different than when elevated by oral supplementation [40,41], and when leptin signaling was reduced by injecting leptin antagonist to neonates (cf. [37,38] and [42,43]. Nevertheless, here we find that αMUPA’s higher leptin surge precedes its attenuation in weight gain, especially in females, thus supporting a causal link between higher leptin surge and lower body weight, both within and among strains.

Several biochemical mechanisms have been suggested for the connection between elevated leptin levels during postnatal development and long-term altered energy homeostasis. These mechanisms include hypothalamic structural changes and changes in expression levels of functional genes. The hypothalamic structural changes include changes in axonal growth, number, and distribution of neurons involved in energy homeostasis [25,26,29], in the ratio of excitatory *vs*. inhibitory synapses of these neurons [27], and in the anatomical location of leptin receptors [28]. The change in expression levels include changes in expression levels of neuropeptides and intracellular signaling peptides related to energy homeostasis in the hypothalamus [29,63], and to improved functionality of brown adipose tissue in leptin-deficient mice [26]. Future studies should explore the presence of any of these mechanisms in the αMUPA mouse model.

Sexual dimorphism in postnatal leptin levels has been reported for some rodent strains [14,36], but not for others [12,35,56,64]. As we show here, and in contrast to their WTs, αMUPA females experience a higher and longer leptin surge than their male littermates. These results confirm our previous results of greater sexual dimorphism in leptin levels of αMUPA mice [45]. Yet, in that study we found lower sexual dimorphism in several parameters of energy and circadian homeostasis of αMUPA mice. In addition, in both the current and the previous [45] studies we found delayed onset of postnatal sexual dimorphism in body weight, as we find here for body composition as well. It may be concluded, therefore, that while uPA overexpression may override sexual dimorphism in energy and circadian homeostasis, that is not the case for leptin production and secretion rates.

### Age-dependent leptin:Fat mass ratio better explains leptin’s postnatal surge than total fat mass

In order to better understand the longer and higher leptin surge in αMUPA mice, we explored the relationship between body fat, and age, strain, and sex. Moreover, we looked at the correlation between total fat mass and leptin at multiple ages before and after the peak, in addition to the more commonly measured time points such as during the peak and at preweaning [13–16]. We used several statistical methods, all showing a weak association between fat mass and leptin when age is not accounted for. This correlation increased substantially when age was included as a cofactor, reflecting the fact that the leptin:FM ratio decreases continuously with age. Specifically, leptin levels of αMUPA females were much higher relative to their fat mass before and during the surge, compared with their wild type as well as their male peers. To our knowledge, this pattern has never been described in the literature before.

Previous studies have shown that leptin is produced and secreted mostly from fat tissues in pups of all rodent strains tested to date [13–16], as well as in fat tissue of mature individuals of our wild type strain, FVB/N [65]. It is therefore safe to assume the same for our αMUPA mice as well. Yet, these studies have also found that postnatal leptin production rate (mRNA expression level in white and brown fat) is a stronger predictor of circulating leptin levels than postnatal fat mass [13,15,16]. Our results support that fat mass in not a strong predictor of postnatal circulating leptin levels and further suggest that leptin ontogeny in both strains and sexes are driven mainly by differences in its production and secretion rates, and that overexpression of uPA protein in αMUPA is responsible for higher production and secretion rates in both pups and lactating dams, independent of fat mass, and possibly outside fat tissues. To further confirm this hypothesis, future studies should aim to detangle age-dependent and tissue-specific changes in leptin’s production and secretion rates, as well as in gastrointestinal absorption rates, both within and between αMUPA and its wild type (see below).

### Maternal-related factors contribute to *αMUPA’s* higher and longer postnatal leptin surge

It has been shown that leptin is produced in the mammary epithelium of humans and rats, and is present in maternal milk [5,18]. However, the effect of maternal milk on pups’ circulating leptin levels, as well as the effect of maternal care per-se, has been debated in the literature [2]. On the one hand, milk leptin was not found to be a determinant of pup’s serum level in rats [21], and the timing of the peak in milk-borne leptin differed than that of the pup’s leptin surge [22]. Moreover, there was no difference in milk-borne leptin levels between dams fed on HFD and regular diet, while leptin levels in their pups was dependent on diet [14]. And finally, leptin was not detectable in leptin-deficient (ob/ob) offspring of ob/+ dams [23]. Other studies, on the other hand, found that rat’s milk-borne leptin peaks at about the same day as the pups’ leptin surge [20]. Moreover, when recombinant leptin was supplied orally to suckling rats, it was absorbed through the stomach and contributed to their total circulating levels [17–19]. Accordingly, maternal deprivation of 4–12 h and 12–24 h led to a decrease in leptin level of P8 mice and P9 rats, respectively [24,55,56]. The decrease in leptin level of P8 mice was only partially mitigated by glucose administration, indicating that leptin reduction due to maternal deprivation is not due solely to lack of nutritional energy [24] but is likely due to the lack of milk-borne leptin as well.

In the current study, we found that leptin is present in the milk of lactating dams from both strains, similarly to findings for other rodents [21,22], but its level is significantly higher in lactating αMUPA dams at the peak of the surge. This higher level in αMUPA dams’ milk was matched by their female pups' serum levels and stomach content under undisrupted conditions. Moreover, 4-hours maternal deprivation decreased serum leptin levels in αMUPA female pup, and eliminated the strain differences found for non-deprived pups. In contrast, four hours of maternal deprivation did not affect serum leptin levels of wild type pups.

These findings suggest that the higher and longer postnatal leptin surge found for non-deprived αMUPA pups is affected by maternal factors, and could be interpreted in at least two most-parsimonious ways. First, αMUPA pups may be more sensitive to maternal deprivation, and particularly to the resulting nutritional shortage and lack of pup-dam interaction, which would indirectly decrease their leptin endogenous production and secretion rates more than in the WT. The results of previous studies and our current study do not support this interpretation. Indeed, maternal deprivation (ranging from 4 to 24 h) during the second week of postnatal development (e.g. P8 to P12 mice) has been shown to be associated with elevated corticosterone levels ([24,56] but see [13])–indicating stress due to the lack of nutrition, pup-dam interaction, and thermoregulation aid, among others. Our results also indicate that 4 hours of maternal deprivation induce a 2–3 fold surge in corticosterone levels. However, the increase in αMUPA pups' corticosterone level was not significantly different from that of the WT. Moreover, previous studies show that αMUPA mice have the same corticosterone levels following 12-h fasting as the WT, indicating again a similar stress responsiveness [44]. αMUPA mature female mice also respond just as fast as their WT to changes in feeding time [66,67], and increase their food intake following injection of leptin antagonist to the same extent as their wild type [68]. Thus, all together, there is no evidence that maternal separation, with its associated restriction of food and interaction, would be more (or less) stressful for αMUPA than for the WT. Yet, we are aware that the aforementioned data is circumstantial and should be further validated while accounting for various factors that are related with endogenous production-secretion rates of leptin.

A second way to interpret the current data is that the elevated leptin level in αMUPA dams contributes directly to pups' serum level via gastrointestinal absorption of milk, as has been suggested previously for other strains [17–19]. However, the experimental setup used here cannot differentiate between: a., direct contribution of the elevated milk-borne leptin to pups serum leptin levels; b., indirect effect of other milk-borne factors that may increase pup’s endogenous leptin production rate; and c., milk-borne factors that increase gastrointestinal absorption of milk-borne leptin. Moreover, our experimental setup cannot prove conclusively that milk-borne leptin is indeed absorbed by αMUPA and WT mice through ingestion, or that there is a strain difference in the gastrointestinal absorption rate of leptin. Therefore, in order to explore these options, future studies will need to test for example gastrointestinal absorption rates of exogenous (labeled) leptin (as in [18,19]), or the effect of maternal deprivation on pups' leptin levels while at the same time measuring stress hormone levels and continually supplying nutritional factors (e.g., synthetic milk or glucose, as in [15,24].

### Perspectives and significance

The findings presented here show that postnatal leptin levels are only related to fat mass during their post-weaning rise. During the pre-weaning leptin surge, on the other hand, they are mainly explained by the age-dependent changes in the leptin:fat mass ratio that may reflect endogenous production rates of leptin and (or) gastrointestinal absorption rates of milk-borne leptin. This study also supports the use of αMUPA as a unique animal model in which a higher and longer postnatal leptin surge is modulated by maternal factors (e.g. high milk-borne leptin level), and is accompanied by greater longevity, lower body-weight, and lower food intake. This animal model can also be utilized for identifying maternal-related sources of higher leptin surge, as well as to relate postnatal leptin’s ontogeny with the ontogeny of energy homeostasis circuits. It is well established that the timing of developmental processes in altricial rodents is postponed in comparison to precocial humans—in which human fetal leptin level increases along the third trimester and rapidly decreases post birth [69]. And yet, similarly to αMUPA mice, higher breast-milk leptin of non-obese human mothers is associated with a lower tendency towards excessive weight gain of their offspring later in life [70]. Therefore, this and future experiments using the αMUPA mouse model could improve our understanding of the mechanisms underlying the ongoing increase in childhood obesity.

## Materials and methods

### Animals, experimental design and data collection

The Experiment was approved by the Israeli Committee for Animal Experimentation. Experiments were conducted in full compliance with the strict guidelines for animal care and use of Tel Hai College, MIGAL, and the Israel Committee for Animal Experimentation. Mice colonies were established from homozygote αMUPA and WT mice, obtained from the Weizmann Institute of Science (Rehovot, Israel), and were held and bred as described previously [45]. Pregnant dams were examined daily to determine day of delivery, designated as postnatal day 0 (P0). Pups were weaned at day 24 (P24), at which point males were separated from females. Litter size was not adjusted because we aimed to measure pups’ leptin levels under the same undisturbed conditions as in previous studies for the sake of comparability [45,52]. Nevertheless, litter sizes of αMUPA and WT only differed by about 1 mouse per litter (WT, 8.5 ± 0.2 vs. αMUPA, 7.3 ± 0.2)[52]. Therefore, although hypothetically possible, it is unlikely that such a small difference in litter size would lead to such large differences in the overall phenotype.

Three experiments were conducted. In the first and second experiment, pups were sampled at 4-day intervals from P4 until P32 (first experiment) or until weaning at P24 (second experiment). At each age-group, data was collected from two to six litters per strain (S2 Table). Each litter was used for only one age-point. For the third experiment we used six P12 litters from each strain. Both sexes were sampled in the first experiments, while for the second and third experiment we used only female pups, which showed greater strain differences in the first experiment. We distinguished sex between PD 4 males and females by the ano-genital distance with males having a greater distance than females [21]. At each age-group, all pups were weighed and a subsample of 1–3 pups from each sex were randomly selected for further analysis (e.g. body composition and serum analysis). In the first experiment, dams were left in the cage but food was removed four hours before blood sampling. By doing so, we controlled for dams’ metabolic state and hence their milk-borne factors, as well as pups’ metabolic state once they started feeding on chow diet. Altricial laboratory mice pups begin to eat solid food only at about P17 and can rely on milk up to ca. P26, and even P28 in large litters. Therefore, we assume that leptin levels of the P4-P24 pups sampled at the first experiment, and more specifically those sampled at about the surge peak, represent an *ad libitum* metallic state. In the second experiment, both dams and food were removed four hours before blood sampling. This manipulation allowed us to explore the effect of maternal (milk-borne) factors on pups’ leptin levels. In the third experiment we measured leptin levels in undisturbed dams and pups.

Body weight measurement, handling, blood sampling following anesthesia, and stomach content sampling following euthanasia were performed between 10:00 to 13:00, as described previously [45]. Mice were anesthetized with isoflurane, and euthanized by an overdose of anesthesia. Leptin level in the serum, milk, and stomach content was measured using a mouse leptin ELISA kit (R&D Systems Inc.), as used previously for both strains [44,45]. Corticosterone level in the serum was measured using a mouse leptin ELISA kit (R&D Systems Inc.), as used previously for both strains [44]. Maternal milk was sampled following [71–73]. Briefly, P12 dams were separated from their pups by placing them, with food and water *ad lib*, in an open glass jar placed in the cage–thus maintaining eye contact between them and their pups and encouraging milk production [71]. Following 4 hours from separation, dams were injected with oxytocin (2 IU/kg, subcutaneous) following [72], and 15 min later dams were milked by hand with the aid of 1 ml syringe, yielding 30 to 60 microliter from all nipples altogether [72,73]. Thereafter pups were rejoined with their mothers and 30 min later were sacrificed, their blood was sampled as mentioned above, and their stomach content was sampled using a needle following cervical dislocation, yielding ca. 50 to 150 microliter [74]. Milk and stomach content were stored as-is at -80°c. Body composition [fat mass (FM), fat-free mass (FFM), and fat mass percentage (Fat %)] were analyzed by full body chemical extraction following [21,75], as in our previous studies [76].

### Statistical analysis

All results are expressed as means ± standard error of the mean (s.e.m). The effect of strain, age, and sex (or maternal deprivation), on body weight, body composition (FM and FFM), corticosterone, and leptin levels were evaluated by two or three-ways ANOVA, followed by the appropriate post hoc analysis. These analyses were done with STATISTICA 7.0 software (StatSoft, Tulsa, OK). Multiple linear regressions were applied to predict the amount of leptin by mice characteristics (strain, gender, age, and FM), due to a higher R2 and lower p-value. Pearson correlations were applied for testing the effects between the continuous variables. The significance of the difference between each two Pearson correlation coefficients was calculated using the Sobel test. The effect of strain and tissue (for ex. maternal serum vs. maternal milk) was evaluated by Mann-Whitney non-parametric test, or Wilcoxon non-parametric test, accordingly. These data were analyzed using the SPSS version 23 (SPSS Inc., Chicago, IL, USA). P value of 5% or less was considered statistically significant.

## Supporting information

S1 Table Influence of strain, sex, and age on body weight, circulating leptin level, fat mass, fat free mass, fat percentage, and leptin: FM ratio during the first experiment; results of three-ways ANOVA for P4 to P32 mice, except for leptin and leptin:FM done for P4 to P24 mice. (DOCX)

S2 Table Number of litters and mice used at each age and experiment for the different measurements. (DOCX)

S3 Table The results of the Pearson correlation between fat mass and leptin, for both strains and genders pulled together. (DOCX)

S4 Table The influence of strain, dams removal, and age on body weight and leptin levels during the second experiment vs. the first experiment; results of three-ways ANOVA for P4 to P24 mice. (DOCX)
