# Supplementary material for: Long-lived weight-reduced αMUPA mice show higher and longer maternal-dependent postnatal leptin surge
Source: PLoS One. 2017 Nov 30;12(11):e0188658. doi: 10.1371/journal.pone.0188658 (PMC5708666; doi:10.1371/journal.pone.0188658)
Supplement: S1 Table — (DOCX) [file pone.0188658.s001.docx]

Table S1: Influence of strain, sex, and age on body weight, circulating leptin level, fat mass, fat free mass, fat percentage, and leptin: fat mass ratio during the first experiment; results of three-ways ANOVA for P4 to P32 mice, except for leptin and leptin: fat mas ratio done for P4 to P24 mice.

| Factor/ Parameter | Body weight | Fat mass | Fat free mass | Fat percentage | Leptin level | Leptin:FM |
| --- | --- | --- | --- | --- | --- | --- |
| Strain | <0.001 | 0.349 | <0.001 | <0.001 | <0.001 | <0.01 |
| Sex | <0.001 | <0.05 | <0.001 | <0.05 | <0.01 | <0.001 |
| Age | <0.001 | <0.001 | <0.001 | <0.001 | <0.001 | <0.001 |
| Strain*Sex | 0.584 | 0.962 | 0.578 | 0.235 | <0.01 | <0.001 |
| Strain*Age | <0.001 | <0.001 | <0.001 | <0.001 | <0.01 | <0.05 |
| Sex*Age | <0.001 | <0.001 | <0.001 | 0.459 | 0.597 | 0.054 |
| Strain*Sex*Age | 0.442 | 0.066 | 0.731 | <0.05 | 0.13 | <0.01 |
