# Supplementary material for: Long-lived weight-reduced αMUPA mice show higher and longer maternal-dependent postnatal leptin surge
Source: PLoS One. 2017 Nov 30;12(11):e0188658. doi: 10.1371/journal.pone.0188658 (PMC5708666; doi:10.1371/journal.pone.0188658)
Supplement: S2 Table — (DOCX) [file pone.0188658.s002.docx]

Table S2: Number of mice used at each age and experiment for the different measurements

| Age |  |  | First experiment | | | | Second experiment | |
| --- | --- | --- | --- | --- | --- | --- | --- | --- |
|  |  |  | WT | | αMUPA | | WT | αMUPA |
|  | Measurement |  | Female | Male | Female | Male | Female | Female |
| 4 | Body weight | No. of Mice | 8 | 7 | 14 | 17 | 8 | 11 |
|  |  | No. of Litters | 3 | 4 | 6 | 5 | 2 | 3 |
|  | Body composition | No. of Mice | 6 | 6 | 14 | 17 |  |  |
|  |  | No. of Litters | 2 | 2 | 6 | 5 |  |  |
|  | Serum leptin | No. of Mice | 6 | 6 | 9 | 14 | 6 | 6 |
|  |  | No. of Litters | 3 | 3 | 4 | 5 | 2 | 3 |
| 8 | Body weight | No. of Mice | 9 | 9 | 9 | 9 | 15 | 17 |
|  |  | No. of Litters | 2 | 3 | 4 | 4 | 3 | 5 |
|  | Body composition | No. of Mice | 9 | 9 | 9 | 7 |  |  |
|  |  | No. of Litters | 2 | 3 | 4 | 3 |  |  |
|  | Serum leptin | No. of Mice | 9 | 7 | 7 | 9 | 10 | 9 |
|  |  | No. of Litters | 2 | 3 | 4 | 3 | 3 | 3 |
| 12 | Body weight | No. of Mice | 8 | 9 | 7 | 9 | 11 | 8 |
|  |  | No. of Litters | 3 | 2 | 3 | 3 | 4 | 2 |
|  | Body composition | No. of Mice | 7 | 9 | 7 | 7 |  |  |
|  |  | No. of Litters | 3 | 2 | 3 | 3 |  |  |
|  | Serum leptin | No. of Mice | 7 | 9 | 7 | 7 | 7 | 7 |
|  |  | No. of Litters | 3 | 2 | 3 | 2 | 3 | 2 |
| 16 | Body weight | No. of Mice | 12 | 8 | 8 | 8 | 9 | 10 |
|  |  | No. of Litters | 3 | 3 | 3 | 3 | 2 | 2 |
|  | Body composition | No. of Mice | 11 | 8 | 7 | 8 |  |  |
|  |  | No. of Litters | 3 | 3 | 3 | 3 |  |  |
|  | Serum leptin | No. of Mice | 10 | 6 | 6 | 8 | 7 | 7 |
|  |  | No. of Litters | 3 | 3 | 3 | 3 | 2 | 2 |
| 20 | Body weight | No. of Mice | 22 | 18 | 16 | 8 | 11 | 9 |
|  |  | No. of Litters | 3 | 5 | 4 | 4 | 3 | 2 |
|  | Body composition | No. of Mice | 22 | 16 | 16 | 8 |  |  |
|  |  | No. of Litters | 3 | 3 | 4 | 3 |  |  |
|  | Serum leptin | No. of Mice | 6 | 12 | 12 | 8 | 7 | 8 |
|  |  | No. of Litters | 2 | 3 | 4 | 3 | 3 | 2 |
| 24 | Body weight | No. of Mice | 11 | 12 | 7 | 7 | 9 | 9 |
|  |  | No. of Litters | 4 | 3 | 2 | 2 | 2 | 4 |
|  | Body composition | No. of Mice | 9 | 9 | 7 | 7 |  |  |
|  |  | No. of Litters | 4 | 2 | 2 | 2 |  |  |
|  | Serum leptin | No. of Mice | 11 | 11 | 7 | 6 | 6 | 8 |
|  |  | No. of Litters | 4 | 3 | 2 | 2 | 2 | 4 |
| 28 | Body weight | No. of Mice | 11 | 8 | 9 | 9 |  |  |
|  |  | No. of Litters | 3 | 4 | 4 | 2 |  |  |
|  | Body composition | No. of Mice | 9 | 8 | 9 | 9 |  |  |
|  |  | No. of Litters | 2 | 4 | 3 | 2 |  |  |
|  | Serum leptin | No. of Mice | 10 | 8 | 9 | 9 |  |  |
|  |  | No. of Litters | 2 | 4 | 3 | 2 |  |  |
| 32 | Body weight | No. of Mice | 7 | 6 | 6 | 7 |  |  |
|  |  | No. of Litters | 2 | 3 | 2 | 5 |  |  |
|  | Body composition | No. of Mice | 7 | 6 | 5 | 7 |  |  |
|  |  | No. of Litters | 2 | 3 | 1 | 5 |  |  |
|  | Serum leptin | No. of Mice | 7 | 6 | 6 | 7 |  |  |
|  |  | No. of Litters | 2 | 3 | 1 | 5 |  |  |
