# Supplementary material for: Long-lived weight-reduced αMUPA mice show higher and longer maternal-dependent postnatal leptin surge
Source: PLoS One. 2017 Nov 30;12(11):e0188658. doi: 10.1371/journal.pone.0188658 (PMC5708666; doi:10.1371/journal.pone.0188658)
Supplement: S3 Table — (DOCX) [file pone.0188658.s003.docx]

Table S3: The results of the Pearson correlation between fat mass and leptin, for both strains and genders pulled together.

|  | Fat mass (gr) | Leptin (ng/mL) |
| --- | --- | --- |
| Fat mass (gr) | - |  |
| Leptin (ng/mL) | 0.173* | - |
| Age (days) | 0.579*** | -0.374*** |
| With mice age as a control variable | | |
| Leptin (ng/mL) | 0.529*** |  |

*p<.05, ***p<.001
