# Supplementary material for: Long-lived weight-reduced αMUPA mice show higher and longer maternal-dependent postnatal leptin surge
Source: PLoS One. 2017 Nov 30;12(11):e0188658. doi: 10.1371/journal.pone.0188658 (PMC5708666; doi:10.1371/journal.pone.0188658)
Supplement: S4 Table — (DOCX) [file pone.0188658.s004.docx]

Table S4: The Influence of strain, dams removal, and age on body weight and leptin levels during the second experiment vs. the first experiment; results of three-ways ANOVA for P4 to P24 mice.

| Factor | Body weight | Leptin level |
| --- | --- | --- |
| Strain | <0.001 | <0.001 |
| Dams removal | <0.001 | <0.001 |
| Age | <0.001 | <0.001 |
| Strain*Dams removal. | 0.714 | <0.001 |
| Strain*Age | <0.001 | <0.001 |
| Dams removal*Age | <0.001 | <0.001 |
| Strain*Dams removal*Age | <0.001 | <0.05 |
